# In Vitro and In Vivo Antihyperglycemic Effects of New Metabiotics from *Boletus edulis*

**DOI:** 10.3390/life14010068

**Published:** 2023-12-30

**Authors:** Anca Zanfirescu, Ionela Avram, Florentina Gatea, Răzvan Roșca, Emanuel Vamanu

**Affiliations:** 1 Faculty of Pharmacy, “Carol Davila” University of Medicine and Pharmacy, Traian Vuia 6, 020956 Bucharest, Romania; anca.zanfirescu@umfcd.ro; 2 Department of Genetics, University of Bucharest, 36–46 Bd. M. Kogalniceanu, 5th District, 050107 Bucharest, Romania; ionela.avram@unibuc.ro; 3 Centre of Bioanalysis, National Institute for Biological Sciences, 296 Spl. Independentei, 060031 Bucharest, Romania; florentina.gatea@incdsb.ro; 4 Anoom Laboratories SRL, 18th Resita Str., ap. 58, 4th District, 024023 Bucharest, Romania; razvan@anoomlaboratories.com; 5 Faculty of Biotechnology, University of Agricultural Sciences and Veterinary Medicine, 011464 Bucharest, Romania

**Keywords:** modulation process, pattern, mouse, simulation

## Abstract

The increasing incidence of diabetes has prompted the need for new treatment strategies, including natural products that reduce glycemia values. This work examined the in vitro and in vivo antihyperglycemic effects of new metabiotics derived from *Boletus edulis* extracts. The metabiotics were obtained from 100% *B. edulis*, and two other products, CARDIO and GLYCEMIC, from Anoom Laboratories SRL, which contain other microbial species related to *B. edulis*. Our in vitro investigations (simulations of the microbiota of patients with type 2 diabetes (T2D)) demonstrated that *B. edulis* extracts modulate the microbiota, normalizing its pattern. The effects were further tested in vivo, employing a mouse model of T2D. The tested extracts decreased glycemia values compared to the control and modulated the microbiota. The metabiotics had positive effects on T2D in vitro and in vivo, suggesting their potential to alleviate diabetes-associated microbiota dysbiosis.

## 1. Introduction

The composition of a person’s gut microbiota shifts as they get older, possibly due to changes in their diet or in how they conduct their daily lives. In recent decades, increased consumption of animal products and dietary fats has significantly impacted human health [1]. These unhealthy lifestyles have been linked to the rising incidence of type 2 diabetes (T2D), highlighting the crucial role of dietary factors in both its onset and progression. Furthermore, a high-fat diet disturbs the gut microbiota, leading to increased inflammation and reduced abundance and diversity of gut microbes in T2D [2,3].

The microbiological characteristics of the microbiota in individuals with T2D exhibit significant variations. Studies have reported inconsistent results, with some finding reduced proportions of the phylum *Firmicutes* and class *Clostridia* in T2D patients compared to healthy controls [4], with these bacteria also being prevalent in obese T2D patients [5]. Another study showed that the T2D microbiota has a high number of Enterobacteriaceae that have a high resistance to modulation compared with the cardiovascular pattern [6]. The complexity of this topic arises from the variability in the gut microbiota across different studies, influenced by many factors. The change in the compositional profile of the gut microbiota and especially the disruption of the eubiosis of the *Bacteroidetes/Firmicutes* phylum have been linked to the appearance and evolution of T2D [7]. At the same time, bacteria such as *Lactobacillus fermentum*, *L. plantarum*, *L. casei*, *Roseburia intestinalis*, and *Akkermansia muciniphila* can prevent and reduce the risk of developing this condition [8]. The dietary fiber intake has been frequently correlated with the evolution of T2D. Fibers represent the main energy source for the gut microbiota and induce the production of SCFAs, which are linked to the blood glucose homeostasis process [9].

Furthermore, as diabetes advances, the number of butyrate-producing bacteria and the production of short-chain fatty acids (SCFAs) by microorganisms decrease. SCFAs are byproducts of anaerobic bacterial fermentation of dietary fibers in the large intestine. Among the several treatment options for modulating the gut microbiota in T2D, oral supplementation with prebiotics seems to have the most beneficial effects, acting through multiple pathways [10,11]. Mushroom polysaccharides serve as fermentable substrates for the gut microbiota, primarily resulting in SCFAs. This decreases the pH of the digestive tract and increases the predominance of health-promoting gut microbiota [12]. Compared with the classical known products (probiotics, prebiotics, psychotics, etc.), metabiotics are a novel functional product that may positively modify the human microbiota fingerprint. Extracts derived from *B. edulis* might be used in this product, which could induce a targeted microbiological and metabolomic modulation in certain groups of populations [12].

The edible mushroom Boletus edulis has both nutritional and medicinal properties. It is rich in carbs, proteins, minerals, and aromatic compounds while having low fat and calorie contents [13,14]. These porcini mushrooms contain bioactive compounds, such as polysaccharides, contributing to their therapeutic properties [15]. Polysaccharides extracted from *B. edulis* using hot water significantly boost the immune function in mice bearing Renca tumors [5,16]. Our recent in vitro experiments using a prebiotic product developed from *Boletus edulis* polysaccharides, conducted in a three-stage human colon simulator system (GIS2), revealed that they could preserve the gut microbiota of individuals with nutritional disorders [17]. Therefore, we hypothesized that the metabiotic product isolated from *Boletus edulis* may also demonstrate protective effects on the microbiota of individuals with T2D. To our knowledge, this is the first study to evaluate the impact of metabiotics on the gut microbiota of individuals with T2D in vivo and in vitro.

## 2. Methods

### 2.1. Vegetal Product and Extraction Process

We tested three obtained functional extracts using a previously described method [18]. Product 1 contained 100% *Boletus edulis* extract [19]. Product 2 was based on a mixture of extracts: *B. edulis* 50%, *Ganoderma lucidum* 25%, *Cordyceps militaris* 15%, *Inonotus obliquus* 10%, and Crataegus monogyna fruits. Product 3 comprised 50% *B. edulis* dried mushrooms, 35% *Auricularia auricula-judae*, 15% *Cordyceps militaris*, and 10% *Aronia melanocarpa* extract.

### 2.2. Analysis of Polyphenolic Compounds via Zonal Capillary Electrophoresis with a Diode Array Detector

Polyphenols (phenolic acids and flavonoids) from each sample and standards were separated using Agilent G7100 capillary electrophoresis apparatus (Agilent Technologies, Ratingen, Germany) equipped with a diode array detector. A standard silica capillary with a 50 µm diameter and an effective length of 63 cm was used for analyte migration. The background electrolyte (BGE), adjusted to a pH of 9.35 with 1 M HCl, consisted of 45 mM sodium tetraborate and 0.9 mM sodium dodecyl sulfate. BGE and all samples were filtered through 0.2 µm membranes (Millipore, PTFE, Bedford, MA, USA) and degassed before use. Migration occurred at 30 °C with an applied voltage of 25 kV and a hydrodynamic sample injection for 10 s at a pressure of 30 mbar. The system was washed with 1 M NaOH for 2 min and rinsed with ultrapure water for 3 min and BGE for 3 min after each 45 min migration. Polyphenols were detected in the 280–360 nm range, with quantification at 280 nm.

Polyphenols standards, including cinnamic acid, chlorogenic acid, sinapic acid, syringic acid, ferulic acid, coumaric acid, caffeic acid, gallic acid, hesperidin, catechin, naringenin, rutin, isoquercetin, quercetin, kaempferol, and myricetin, all of analytical purity >98%, were purchased from Sigma-Aldrich (Saint Louis, MO, USA). Polyphenolic compounds were identified by comparing the retention times and standard addition [20].

### 2.3. In Vitro Modulation of D2T-Associated Microbiota

Following a previously published method, the in vitro simulation was performed using a GIS1 single-chamber simulator [19]. Multiple microbiotas sourced from donors (age range: 45 to 77, both men and women in equal numbers) who had refrained from taking medications that might influence microbiota patterns for the preceding six months were used for in vitro tests and to determine the modulatory effect of metabiotics.

The microbiotas were obtained from the ColHumB Collection of the Laboratory of Pharmaceutical Biotechnologies, UASVM Bucharest (www.gissystems.ro). The experimental methodology followed the ethical criteria of the UASVM Bucharest (ColHumB Registration number: 1418/23 November 2017 [21]). The microbiota samples, which reflect the number of microorganisms, were preserved in a freezer using a solution of 20% glycerol until they were ready to be analyzed. The mixture produced after the simulation was stored in the freezer for the study of organic acids and microbiological patterns.

Each sample was thawed, and 1 mL was used for DNA extraction using a PureLink Microbiome DNA Purification Kit (Invitrogen, Waltham, MA, USA). Each reaction included Power SyberGreen PCR Master Mix 2× (Applied Biosystems, Waltham, MA, USA) and 5 ng of DNA. The remaining PCR amplification conditions, including specific information on the primers, were previously documented in another work [22].

### 2.4. Analysis of SCFAs Produced Following In Vitro Microbial Modulation

The separation of SCFAs was achieved using reverse polarity zonal electrophoresis [23] using Agilent G7100 capillary electrophoresis equipment (Agilent Technologies, Ratingen, Germany), which was fitted with a diode array detector and a standard silica capillary (50 µm diameter, with an effective length of 63 cm). The migration conditions were as follows: temperature 25 °C, a voltage of −20 Kv, hydrodynamic injection for 10 s at 35 mbar, detection at λ = 200 nm, and a migration buffer comprising H_3_PO_4_ 0.5 M, cetyltrimethylammonium bromide (CTAB) 0.5 mM at pH = 6.24 (adjusted with NaOH), and 15% methanol. The capillary was washed between two separations with 1 M NaOH for 2 min, ultrapure water for 2 min, and BGE for 3 min. Standard solutions were prepared in water, stored at +4 °C, and diluted daily. SCFAs were identified by comparing the retention times and standard addition.

All samples were filtered using a 0.2 μm filter (MilliporeSigma, Bedford, MA, USA) and degassed before use. All utilized reagents were of analytical purity (>98%). Succinic, formic, isovaleric, benzoic, 3-(-4-hydroxyphenyl) lactic, phenyl lactic, and propionic acids were purchased from Sigma-Aldrich (USA); butyric and DL-lactic acids were purchased from Fluka (Buchs, Switzerland); acetic acid was procured from Riedel-de-Haën (Germany); oxalic and phosphoric acids 85% were purchased from Merck (Germany); CTAB was procured from Loba Chemie (Austria); and 0.1 N and 1 N NaOH, as well as water of chromatographic purity, was procured from Agilent Technologies (Santa Clara, CA, USA).

### 2.5. In Vivo Effect of Administration of Functional Mushroom Extracts

To determine the antidiabetic and hypolipidemic actions of functional mushroom extracts (P1, P2, and P3), a group of 52 male Sprague Dawley rats from the Cantacuzino biobase was used. The animals were kept in quarantine for three days, after which they were housed in a ventilated cage system with free access to water and pelleted food. The temperature and relative humidity were kept constant throughout the experiment (22–24 °C, 45–60%) using a hygrometer. All procedures complied with the Directive 2010/63/EU which came into force on 1 January 2013. The protocol was designed based on ARRIVE guidelines and was approved by the Ethics Committee of the University of Medicine and Pharmacy Bucharest (no. 0045/2020).

Diabetes was induced using one intraperitoneal injection of alloxan at 130 mg/kgc. Before the administration, the animals were kept without food for 24 h. After 48 h, the blood glucose levels were determined using a Codefree glucometer. Diabetic rats were randomly assigned into 5 groups (*n* = 8) and received the following treatments: group II, physiological serum 0.1 mL/100 g per os (p.o.); group III, metformin 100 mg/kg p.o.; group IV, P1 12 mL; group V, P2 12 mL; group VI, P3 12 mL. Additionally, a control group of mice with normal blood glucose values (<120 mg/100 mL), group I, was administered 0.1 mL/100 g saline. The duration of the treatment was 12 days. Glycemia and body weights were determined on days 1, 3, 6, 9, and 12 after the induction of diabetes. Blood was collected for further testing on day 12, 2 h after administration.

### 2.6. Assessment of Oxidative Stress in Rat Plasma

The assessment of oxidative stress levels in plasma involved the quantification of lipid peroxides and carbonylated protein concentrations. Lipid peroxides were analyzed using the thiobarbituric acid reactive substances method [24], and the results were expressed in nmol of malondialdehyde (MDA) per mL of plasma. Carbonylated proteins, resulting from the interaction between reactive oxygen species and proteins, were evaluated in the plasma using the guanidine hydrochloride method [25], with the results expressed in nmol per milligram of protein.

### 2.7. Statistical Analysis

The parameters were assessed three times, and the outcomes are presented as the average values plus or minus the standard deviation (SD). Statistical analyses were performed using the IBM SPSS Statistics 23 software program (IBM Corporation, Armonk, NY, USA). An analysis of variance (ANOVA) was used to evaluate differences between groups, followed by Tukey’s post hoc analysis. The experimental data were analyzed and correlated using the IBM SPSS Statistics software program (IBM Corporation, Armonk, NY, USA). The significance level for the computations was established as follows: for a significance level of *p* < 0.05, the result was considered significant; for a significance level of *p* < 0.01, the result was considered very significant; for a significance level of *p* < 0.001, the result was considered highly significant; and for a significance level of *p* < 0.0001, the result was considered very significant. These levels are denoted by the letters a to d.

## 3. Results

### 3.1. Phenolic Compound Composition in the Samples

An analysis of the three extracts showed that polyphenolic compounds are present in significant amounts in all the samples, as shown in Table 1. Rutin, catechin, and hesperidin were found in the highest concentrations, with smaller amounts of chlorogenic acid, ferulic acid, naringenin, sinapic acid, and syringic acid detected. These polyphenols, along with other compounds, bolster antioxidant support and collectively influence microbiota responses and metabolic processes [26].

### 3.2. In Vitro Effect of the Tested Extracts on Dysbiotic Microbiota

Results of the in vitro molecular analysis of the microbiota of individuals with T2D are shown in Table S1. Research on the gut microbiota composition in people with T2D has focused on broad categories of bacteria. Herein, compared to CARDIO, the GLYCEMIC product positively impacted the microbial pattern, which was associated with a gradual improvement in dysbiosis and a decrease in insulin resistance (Table 2). Changes in the relative abundance of *Firmicutes* and *Bacteroidetes* and the *Firmicutes/Bacteroidetes* ratio were balanced following treatment with GLYCEMIC. Gum arabic (control) exhibited limited action due to the lack of a carbon source.

From a metabolic point of view, the levels of organic acids (Table 3) were associated with changes in the microbiota. The administration of P3 increased the concentration of some SCFAs, particularly that of acetic and propionic acids, which more than doubled compared to the control, while the level of butyric acid exhibited only a slight increase. Similar trends were observed following the administration of P1 and P2. An increase in butyrate concentration is associated with improved insulin response, while an increase in propionate levels is associated with stable glucose homeostasis and an increase in glucose-stimulated insulin release [1].

GLYCEMIC administration led to a significant overabundance of lactic acid-producing bacteria (10,040 ± 0.124 g/L), approximately three times higher than the levels in the control group. Gut microbiota dysbiosis can impact metabolic processes and is directly associated with T2D. Our in vitro study revealed notable features, such as the presence of 3-(4-hydroxyphenyl) lactic acid, as potential biomarkers of the effect of GLYCEMIC, influenced by its interaction with gum arabic. As far as we know, this is the first study to report such a result. Another biomarker associated with the response to CARDIO was phenyllactic acid. The metabolism of phenyllactic acid in the intestine may influence the host’s metabolism and intestinal homeostasis. Furthermore, phenyllactic acid has antimicrobial properties. Thus, CARDIO induces distinct microbiota changes compared to GLYCEMIC, indicating its distinct therapeutic effects. 

### 3.3. In Vivo Effect of the Tested Extracts on Glycemia and Oxidative Stress

In the non-diabetic control group, blood glucose values remained relatively constant and normal throughout the experiment (average glycemia at day 12 = 108.88 ± 10.16 mg/100 mL, *p* > 0.05 vs. day 1). A similar trend was observed for the positive diabetic control, where the blood glucose values remained relatively constant after 12 days (average glycemia at day 12 = 564.29 ± 58.15 mg/100 mL). The groups treated with the antidiabetic reference (metformin) and those treated with P3 exhibited a significant decrease in blood glucose levels compared to baseline on all test days (Table 4).

Animals treated with metformin, P2, and P3 exhibited significantly lower blood glucose levels compared to the diabetic control group after 3, 6, 9, and 12 days of administration. Among the tested extracts, P3 had the most pronounced blood-sugar-lowering effect. Furthermore, the tested compounds also reduced the levels of lipid peroxides and carbonylated protein concentrations in diabetic rats (Table 5).

### 3.4. In Vivo Effect of the Tested Extracts on Rat Microbiota

**Figure 1:**
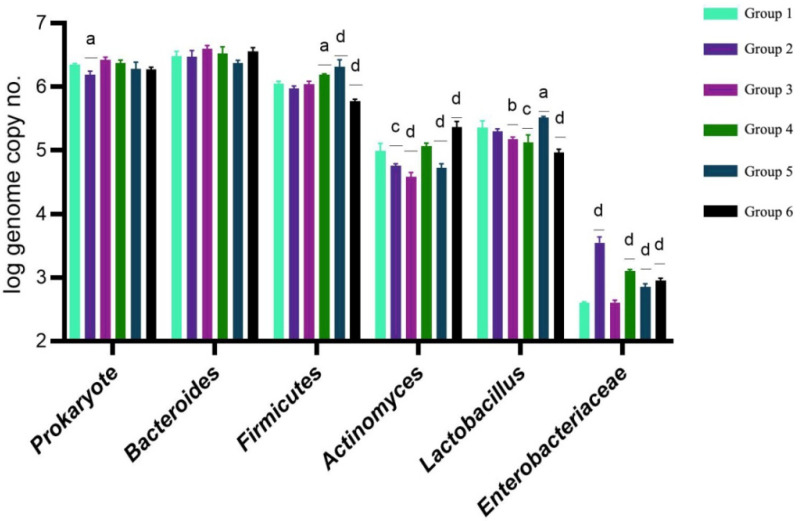
Molecular analysis of the rat microbiota after treatment administration. Control (group 1) vs. samples; a—*p* < 0.05; b—*p* < 0.01; c—*p* < 0.001; d—*p* < 0.0001; *n* = 3.

Following the administration of the tested compounds, the experimental groups exhibited distinct microbial compositions. The levels of Prokaryotes and *Bacteroides* were relatively balanced. A comparative study revealed a unique microbial fingerprint associated with T2D and metformin treatment (Figure 1).

*Enterobacteriaceae* levels varied characteristically, indicating a specific metabolomic fingerprint linked to inflammatory proliferation and dysbiosis [27]. The groups with hyperglycemia exhibited a high abundance of *Bacteroides* and *Firmicutes* and a low abundance of *Lactobacillus*. A comparative analysis of the microbiota at the end of the study (Figure 1) revealed important changes in microbiota patterns, indicating dysbiosis in the diabetic group. No significant differences were observed between the microbiota of the control and metformin-treated groups.

Microbial diversity decreased in T2D groups treated with metformin compared to those treated with the three mushroom extracts. *Bacteroides* levels remained relatively stable. Groups IV, V, and VI exhibited an increase in the abundance of *Lactobacillus*, indicating an amelioration in hyperglycemia-induced dysbiosis, which indicates the efficacy of the tested compounds.

## 4. Discussion

Mushrooms are a rich source of valuable compounds with various biological effects, including antioxidant and anti-inflammatory effects [28]. Additionally, their composition of multiple compounds with synergistic effects modulates the colon microbiota and positively impacts the human body’s homeostasis [2].

While the main effects of medicinal mushrooms are attributed to the beta-glucan content, polyphenols can enhance the antioxidant effect and restore dysregulated metabolic processes [18,29,30,31]. All the extracts tested herein contain rutin in significant amounts, which can partly explain their antidiabetic and microbiota-modulating properties. These results align with those of previous research, demonstrating that rutin modulates the gut microbiota, alleviating hyperglycemia and preventing diabetes-related colon lesions [32] by increasing the abundance of beneficial microbiota such as *Akkermansia* and decreasing the abundance of diabetes-related microorganisms such as *Escherichia* and *Mucispirillum*.

With the emerging trends in human nutrition and the introduction of products such as postbiotics [3], we can now categorize biofunctional products derived from medicinal and/or edible mushrooms as a novel category of metabiotics. The term “metabiotic” is derived from the Greek words “meta” (meaning beyond) and “biotic” (of or relating to living organisms). Our products, composed of bioactive extracts from the mushroom basidiome, contain various compounds, including polysaccharides (β-glucans) and phenolic compounds [11]. It is essential to distinguish them from parabiotics, postbiotics, or probiotics [12]. In particular, paraprobiotics lack any probiotic biomass components. Meanwhile, metabiotics contain both nutritional factors and substances with antimicrobial, anti-inflammatory, antioxidant, and immunostimulatory effects. These effects are partly generated through the modulation of the microbiota’s composition [33] and metabolic activity [12] and impact the homeostasis of the human body [4].

This indirect effect remains highly stable and does not directly depend on the type of product administered; instead, it impacts the body’s physiological functions. This stability is expected to enhance the body’s ability to respond to exogenous and endogenous factors that potentiate oxidative stress [13,34]. Our results indicate that P2 and P3 significantly increase the prevalence of beneficial strains belonging to the genus *Lactobacillus* over time. This increase is a direct result of the presence of β-glucans and their associated proteins, which serve as a food source for certain *Bacteroides* species [35], such as *B. cellulosilyticus* WH2. These bacteria degrade mycoprotein-derived β-glucans, releasing oligosaccharides into the environment. These oligosaccharides are used by other gut microorganisms such as *Lactiplantibacillus* and *Bifidobacterium* [2]. Furthermore, a positive modulation effect was observed, indicating that these strains could alleviate the adverse consequences of increased blood glucose levels. This correlation implies that the metabolomic products and targeted microbial strains can be biomarkers for monitoring glycemic management.

The processes through which metabiotic products positively modulate blood glucose levels raise substantial questions. Additional investigations are required to comprehensively understand the precise mechanisms by which these strains impact glucose metabolism. Moreover, the modulatory potential of metabiotics might provide a means for implementing tailored strategies for diabetes treatment.

While the concept of metabiotics has been discussed in the past [14], this study goes beyond simply obtaining by-products from mushrooms. Certain components possess well-defined characteristics, including an anticipated biological impact. The current research proposes using metabiotic products, including bioactive mushroom extracts with modulatory properties, to treat T2D [25]. This study marks the first ever validation of the antihyperglycemic and microbiota-modifying effects of novel functional metabiotics using in vitro and in vivo experiments. Correlation between in vitro and in vivo data is essential for developing complementary therapies for T2D. Using in vitro and in vivo microbiota modulation tests in T2D validated the potential use of the tested product as a therapeutic intervention. Successful in vitro findings, supported by in vivo evidence, may lead to clinical trials and potential diabetes treatments [36]. Integrating both studies is crucial for effective approaches to manipulating the gut microbiota and mitigating T2D progression [37]. Glucose level data were essential to demonstrate the efficacy of GLYCEMIC and CARDIO in reducing high glucose levels. Additionally, we should highlight that the reduction in glycemic levels is closely associated with cardiovascular disorders [38]. Correlating the effects of our tested extracts on blood glucose levels and cardiovascular risk reduction represents a future research direction.

Preserving the microbiota involves ensuring the composition and equilibrium of gut microbes, often to maintain good health. In contrast, modifying the microbiota composition is conducted to achieve a certain therapeutic outcome, which may be advantageous in illnesses like type 2 diabetes. Diet can function as an additional component in influencing the microbiota. Diet has a well-established influence on the makeup and function of the gut microbiota, making it a crucial factor in modulation techniques. Dietary components, namely those found in *B. edulis*, may directly impact the microbiota, leading to the reported results [39].

The human gut microbiota, a specific pattern of bacteria inhabiting the gastrointestinal system, is profoundly impacted by many variables, including food. The selection of foods we consume significantly influences the structure and performance of the microbiota. Various dietary patterns, such as those rich in fiber, protein, or lipids, may result in unique microbiotic compositions. For instance, a fiber-rich diet promotes the proliferation of advantageous bacteria that metabolize fiber into short-chain fatty acids, resulting in excellent health outcomes [40]. Conversely, diets rich in fat and sugar might stimulate the proliferation of less advantageous bacteria and perhaps contribute to the development of metabolic diseases. In addition to eating, the microbiota is influenced by other variables, including lifestyle, drug use (particularly antibiotics), age, and genetics [41,42]. The intricate interplay of nutrition, lifestyle variables, and the microbiota is a crucial field of study, owing to its potential ramifications for human health, including controlling metabolic or chronic illnesses, gastrointestinal well-being, and even psychological states. Understanding these interactions is crucial for formulating dietary guidelines and therapies to enhance the gut microbiota for improved health outcomes [42].

The effects of extracts derived from medicinal mushrooms on the gut microbiota in situations such as dysbiosis, diabetes, or cardiovascular disorders have been investigated. Researchers have found that polysaccharides from Auricularia auricula-judge (AAP) increase the variety of microbes in the colon of mice with type 2 diabetes (T2D) by increasing the number of genera like Lactobacillus and Bacteroides and decreasing the number of genera like Clostridium and Allobaculum. These polysaccharides regulate the AKT and AMPK pathways to exert their effects in type 2 diabetes [43]. Other research demonstrated that this substance (AAP) augments the functions of the enzymes responsible for neutralizing free radicals and diminishing oxidative stress. Simultaneously, AAP impacts the microbiota by reducing the prevalence of taxa such as Desulfovibrio, Enterorhabdus, and Helicobacter [44]. Extracts from Auricularia auricula-judge not only exhibit hypoglycemic effects but also lower total cholesterol and LDL cholesterol levels, block alpha-amylase, boost liver glycogen and glutathione levels, stimulate plasma C-peptide, and promote GLP-1 secretion [45,46,47]. Effects similar to AA extracts have also been reported for Cordyceps militaris (CM) [48,49,50]. After six weeks of dosing, the CM extract alters the microbiome’s composition in diabetic mice, promoting the proliferation of certain genera. The microorganisms mentioned include Parabacteroides, Eubacterium xylanophilum, Colidextribacter, Roseburia, and Alloprevotella [48]. Administering a blend of Cordyceps militaris fruiting bodies and mycelia powder to diabetic mice reduced glucose levels and normalized triglyceride and cholesterol levels [49]. The efficacy of CM in ameliorating metabolic syndrome is contingent upon the specific compounds present in the supplied extract. Therefore, the polysaccharides derived from CM show superior efficacy in reducing blood glucose and cholesterol levels in mice fed a high-fat/high-sucrose diet, compared to the administration of fruiting bodies or cordycepin. The presence of these polysaccharides promoted the proliferation of the Akkermansia muciniphila population and ameliorated intestinal dysbiosis [50].

The presence of imbalanced and unhealthy gut microbiota may have a role in the development and progression of cardiovascular illnesses via many mechanisms. One such mechanism involves the maintenance of the intestinal barrier’s integrity by microorganisms. This process relies on specific bacterial endotoxins, which are compounds found in the structure of bacteria or their metabolites, entering the bloodstream. These endotoxins can trigger an immune response and cause systemic inflammation [51]. The microbiota also plays a role in the formation of trimethylamine N-oxide (TMAO), a metabolite derived from trimethylamine (TMA). TMA is formed when the gut bacteria act on dietary choline and phosphatidylcholine [52]. Elevated levels of TMAO in the bloodstream are associated with cardiovascular illnesses, and higher TMAO concentrations are correlated with an increased risk of mortality. The bacteria responsible for TMAO production are classified under Anaeroplasmataceae, Prevotellaceae, Deferribacteraceae, and Enterobacteriaceae [53]. TMAO also plays an important role in the occurrence of type 2 diabetes [54,55,56]. Polysaccharides derived from GL have a beneficial impact on the *Firmicutes* to *Bacteroidetes* ratio, leading to a reduction in the populations of Lactobacillus reuteri and Bifidobacterium pseudolongum while favoring the populations of Bacteroides acidifaciens and Alistipes finegoldii [56]. In a separate investigation, an aqueous extract of GL micelles was supplied to mice on a high-fat diet. This resulted in a reduction in inflammation by reducing the levels of TNF-α, IL-1β, and IL-6 proteins, therefore reversing dysbiosis. Administering GL micelle aqueous extracts to mice fed a high-fat diet effectively lowers inflammation by decreasing TNF-α, IL-1β, and IL-6 proteins, correcting dysbiosis. This extract has a significant anti-obesity effect by reducing the populations of the Proteobacteria phylum at the microbiome level [57].

Polysaccharides derived from Inonotus obliquus (IO), a fungus with traditional medicinal usage, provide several beneficial properties, including anti-inflammatory, antioxidant, immunomodulatory, hypoglycemic, hypolipidemic, hepatoprotective, and antioxidant effects [58]. When administered to mice with chronic pancreatitis, these substances affect the activity of crucial enzymes such as glutathione peroxidase, lipase, and trypsin, as well as tumor necrosis factor-alpha and transforming growth factor beta. These chemicals exert their effect at the microbiota level by promoting the growth of *Bacteroidetes* populations and reducing the abundance of *Firmicutes*, thus impacting the variety and overall health of the microbiota [59].

## 5. Conclusions

This research reveals significant variations in gut microbiota compositions across different groups, potentially influenced by factors such as study design, concomitant diseases, and laboratory settings. A comparative analysis between patients with T2D who have not been treated with metformin and those with normal blood glucose levels highlighted T2D-induced microbiota alterations. The current investigation confirms that metformin induces changes in the gut microbiota’s taxonomic composition and functional capacity. Our products have the potential to modulate the intestinal microbiota, contributing to the maintenance of its healthy balance.

## Figures and Tables

**Table 1:** Phenolic compound contents in P1, P2, and P3.

No.	Compound	P1 mg × gDW^−1^	P2 mg × gDW^−1^	P3 mg × gDW^−1^
1	Hesperidin	17.97 ± 0.62	6.43 ± 0.16	18.70 ± 0.88
2	Catechin	83.94 ± 0.14	41.90 ± 0.43	31.34 ± 0.83
3	Naringenin	17.08 ± 0.17	2.20 ± 0.20	1.127 ± 0.07
4	Rutin	311.48 ± 3.44	357.48 ± 8.88	466.52 ± 5.45
5	Cinnamic acid	6.83 ± 0.06	1.99 ± 0.118	1.13 ± 0.07
6	Chlorogenic acid	9.24 ± 0.17	2.31 ± 0.191	2.01 ± 0.06
7	Sinapic acid	22.80 ± 0.11	-	2.71 ± 0.10
8	Syringic acid	8.10 ± 0.10	13.71 ± 0.339	8.64 ± 0.65
9	Ferulic acid	7.22 ± 0.04	3.09 ± 0.225	5.37 ± 0.21
10	Myricetin	3.39 ± 0.02	1.07 ± 0.021	3.83 ± 0.03
11	Caffeic acid	-	2.24 ± 0.19	-
12	Quercetin	-	1.44 ± 0.01	-

**Table 2:** Major genus abundance (log CFU/mL) after in vitro simulations (administration of 230 g compound/day).

Samples	Prokaryote	*Enterobacteriaceae*	*Firmicutes*	*Lactobacillus*	*Actinomyces*	*Bacteroides*
Control—untreated microbiota	8.10 ± 0.01	4.97 ± 0.12 ^b^	7.82 ± 0.05	7.39 ± 0.04	3.74 ± 0.04	3.00 ± 0.42
Control—gum arabic	8.10 ± 0.05	2.74 ± 0.25 ^b^	7.48 ± 0.05 ^b^	7.74 ± 0.07 ^b^	2.15 ± 0.31 ^a^	1.86 ± 0.04 ^a^
P1—ColonX	8.10 ± 0.23	4.96 ± 0.49	7.61 ± 0.006 ^a^	7.98 ± 0.006 ^c^	2.53 ± 0.05 ^b^	3.14 ± 0.04
P2—CARDIO	8.10 ± 0.04 ^a^	3.32 ± 0.08 ^b^	7.41 ± 0.004 ^b^	7.46 ± 0.01	1.51 ± 0.04 ^c^	2.40 ± 0.04
P3—GLYCEMIC	8.03 ± 0.06	4.90 ± 0.07	7.49 ± 0.08 ^a^	7.80 ± 0.03 ^c^	2.99 ± 0.06 ^c^	3.76 ± 0.02

Control vs. sample ^a^—*p* < 0.05; ^b^—*p* < 0.01; ^c^—*p* < 0.001; *n* = 3.

**Table 3:** Concentrations of short-chain fatty acids after in vitro simulations.

Sample	Formic Acidg L^−1^	Oxalic Acidg L^−1^	Succinic Acid g L^−1^	Acetic Acid g L^−1^	Propionic Acid g L^−1^	Lactic Acid g L^−1^	Butyric Acid g L^−1^	Benzoic Acid g L^−1^	Isovaleric Acid g L^−1^	Phenyl Lactic Acid g L^−1^	3-(-4-hydroxyphenyl) Lactic Acid g L^−1^
Control—untreated microbiota	0.428 ± 0.020	-	0.177 ± 0.009	0.436 ± 0.010	0.021 ± 0.002	3.688 ± 0.049	0.023 ± 0.001	-	0.545 ± 0.015	-	-
Control—gum arabic	0.198 ± 0.012	-	0.100 ± 0.011	3.382 ± 0.045	0.075 ± 0.004	9.991 ± 0.086	0.134 ± 0.003	-	4.440 ± 0.051	-	0.012 ± 0.001
P1—ColonX	0.301 ± 0.013	-	0.083 ± 0.009	0.944 ± 0.054	0.065 ± 0.005	11.695 ± 0.113	0.041 ± 0.002	-	1.004 ± 0.011	-	-
P2—CARDIO	0.374 ± 0.003	0.024 ± 0.002	0.079 ± 0.009	1.402 ± 0.013	0.119 ± 0.011	9.216 ± 0.105	0.062 ± 0.004	-	0.982 ± 0.120	0.024 ± 0.003	-
P3—GLYCEMIC	0.323 ± 0.010	-	0.080 ± 0.009	1.117 ± 0.026	0.084 ± 0.009	10.040 ± 0. 124	0.033 ± 0.001	-	0.748 ± 0.123	-	0.020 ± 0.001

**Table 4:** Antihyperglycemic effect of tested extracts compared to diabetic control (%).

Determination Day	Metformin	Product 1 (ColonX)	Product 2 (CARDIO)	Product 3 (GLYCEMIC)
Day 1	−2.00	2.69	1.64	1.15
Day 3	−56.70 *	−4.37	−26.40 *	−28.27 *
Day 6	−68.45 *	−9.19	−22.79 *	−30.08 *
Day 9	−73.71 *	−11.05	−26.20 *	−34.41 *
Day 12	−79.09 *	−12.78	−26.81 *	−35.65 *

*, *p* < 0.05, Student’s *t* test, confidence interval 95%.

**Table 5:** The effect of tested extracts on the oxidative stress parameters.

	Normal	Diabetic	Metformin	P1	P2	P3
Plasma TBARS (nmol/mL)	1.87 ± 0.98	2.75 ± 1.67 ^a^	1.98 ± 0.53 ^b^	2.53 ± 0.78 ^a^	2.38 ± 1.22 ^a^	2.01 ± 0.89 ^b^
Plasma CP (nmol/mg protein)	0.44 ± 0.28	2.58 ± 0.98 ^a^	0.62 ± 0.12 ^b^	2.43 ± 1.43 ^a^	2.6 ± 0.76 ^a^	1.34 ± 0.44 ^b^

Legend: TBARS, thiobarbituric acid reactive substance; CP, carbonylated proteins; ^a^, *p* < 0.05 vs. normal group; ^b^, *p* < 0.05 vs. diabetic group (ANOVA).

## Data Availability

All data generated or analyzed during this study are included in this published article (and its supplementary information files).

## Supplementary Materials

The following supporting information can be downloaded at: https://www.mdpi.com/article/10.3390/life14010068/s1, Table S1: The value of major genous after in vitro simulations.
