# Supplementary material for: In Vitro and In Vivo Antihyperglycemic Effects of New Metabiotics from Boletus edulis
Source: Life (Basel). 2023 Dec 30;14(1):68. doi: 10.3390/life14010068 (PMC10817235; doi:10.3390/life14010068)
Supplement: Supplementary file 1 [file life-14-00068-s001.zip › life-2796162-supplementary.pdf]

Table S1. The value of major genous after *in vitro* simulations

Samples	Prokaryote	Enterobacteriaceae	Firmicutes	Lactobacillus	Actinomyces	Bacteroides
**Control - untreated microbiota**	8.10±0.01	4.97±0.12^{b}	7.82±0.05	7.39±0.04	3.74±0.04	3.00±0.42
**Control - arabic gum**	8.10±0.05	2.74±0.25^{b}	7.48±0.05^{b}	7.74±0.07^{b}	2.15±0.31^{a}	1.86±0.04^{a}
**P1 - ColonX**	8.10±0.23	4.96±0.49	7.61±0.006^{a}	7.98±0.006^{c}	2.53±0.05^{b}	3.14±0.04
**P2 - CARDIO**	8.10±0.04^{a}	3.32±0.08^{b}	7.41±0.004^{b}	7.46±0.01	1.51±0.04^{c}	2.40±0.04
**P3 - GLYCEMIC**	8.03±0.06	4.90±0.07	7.49±0.08^{a}	7.80±0.03^{c}	2.99±0.06^{c}	3.76±0.02
